# Thermal sensitivity analysis data utilizing Q10 scanning, Boltzmann slope factor and the change of molar heat capacity

**DOI:** 10.1016/j.dib.2016.01.025

**Published:** 2016-01-22

**Authors:** KyeongJin Kang

**Affiliations:** Department of Anatomy & Cell Biology, Samsung Biomedical Research Institute, Sungkyunkwan University School of Medicine, Republic of Korea

**Keywords:** Boltzmann slope factor, Q10 scanning, Molar heat capacity, Thermal sensitivity, Infrared, TRPA1

## Abstract

As a further elaboration of the recently devised Q10 scanning analysis (“Exceptionally high thermal sensitivity of rattlesnake TRPA1 correlates with peak current amplitude” [1]), the interval between current data points at two temperatures was shortened and the resulting parameters representing thermal sensitivities such as peak Q10s and temperature points of major thermosensitivity events are presented for two TRPA1 orthologues from rattlesnakes and boas. In addition, the slope factors from Boltzmann fitting and the change of molar heat capacity of temperature-evoked currents were evaluated and compared as alternative ways of thermal sensitivity appraisal of TRPA1 orthologues.

**Specifications Table**

**Table:** 

Subject area	*Biology*
More specific subject area	*Biophysics and electrophysiology*
Type of data	*Graph and figure*
How data was acquired	*Electrophysiology on TRPA1-expressing frog oocytes (TEVC)*
Data format	*Analyzed*
Experimental factors	*Temperature elevation on TRPA1 cRNA injected oocytes*
Experimental features	*Recorded temperature-induced currents were processed by a variation of Q10 scanning or fitted to equations to acquire thermosensitivity-associated parameters.*
Data source location	*Suwon, South Korea*
Data accessibility	*Data are with this article*

**Value of the data**

• Temperature-dependent activation of ion channels is typically quantitated by thermal coefficient Q10.
• For ion channels with fast change of Q10 over varying temperatures, Q10 scanning is useful and further optimized in the data set given here.
• Q10 scanning is improved to provide higher sensitivity for detection of maximal Q10s and more precise temperature parameters such as the temperature points yielding Q10 trace deflection and the maximum Q10.
• The Boltzmann slope factor and the change of molar heat capacity of TRPA1s presented here have not been examined elsewhere for the purpose of comparing thermal sensitivities of ion channels, and are found to reflect difference in thermal sensitivity among thermally sensitive ion channels.

## 1 Data

**Fig. 1:**
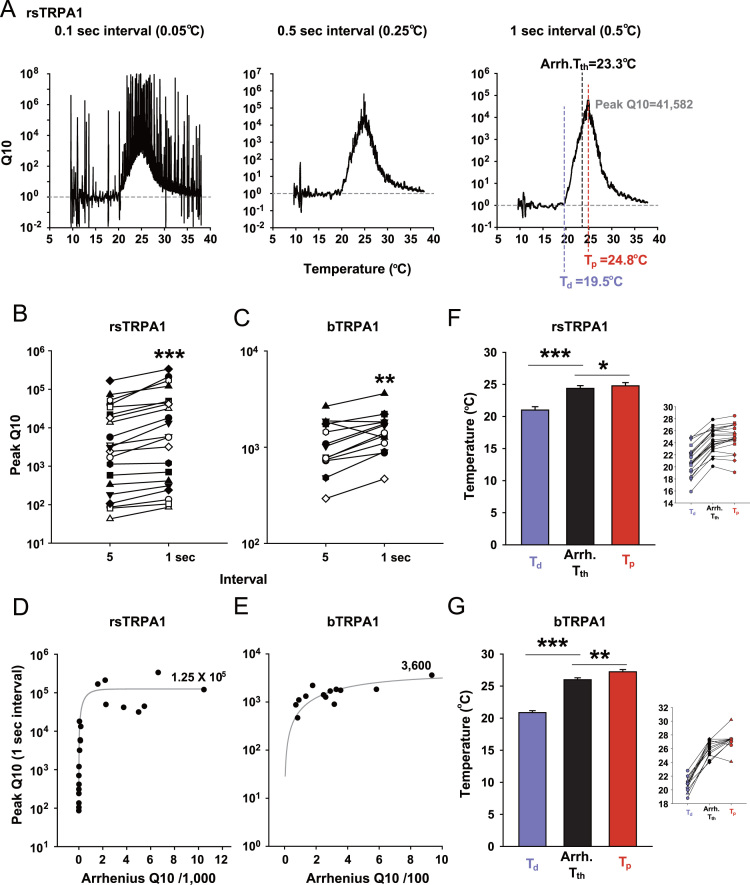
Refining Q10 scanning by noise reduction. (A) Q10 scanning results with the three indicated data intervals. (B and C) Comparison of the peak Q10s between 5-s and 1-s intervals for rsTRPA1 (B) and bTRPA1 (C). The former interval was used in Ref. [1] without Gaussian filtering at 1 Hz. D and E, The new peak Q10s with 1-sec interval were plotted with the Arrhenius Q10 [1] and fitted to the following equation of “exponential rise to maximum” for rsTRPA1 (D) and bTRPA1 (E). *y*=a(1−*e*^−bx^), *a*=123,129.5 and *b*=0.0017 for rsTRPA1. *a*=3,618 and *b*=0.002 for bTRPA1. (F and G) Deflecting temperature (*Td*) and peak temperature (*Tp*) are illustrated in the right panel of (A) in comparison with the Arrhenius threshold temperature (*T*th). The average values of *Td* and *Tp* are presented with *Tth* for rsTRPA1 (F) and bTRPA1 (G). The insets are provided for individual data presentation. *: *p*<0.05, **: *p*<0.01, and ***: *p*<0.001. Paired *t*-test in B and C. ANOVA Repeated Measures, Holm–Sidak test in F and G.

**Fig. 2:**
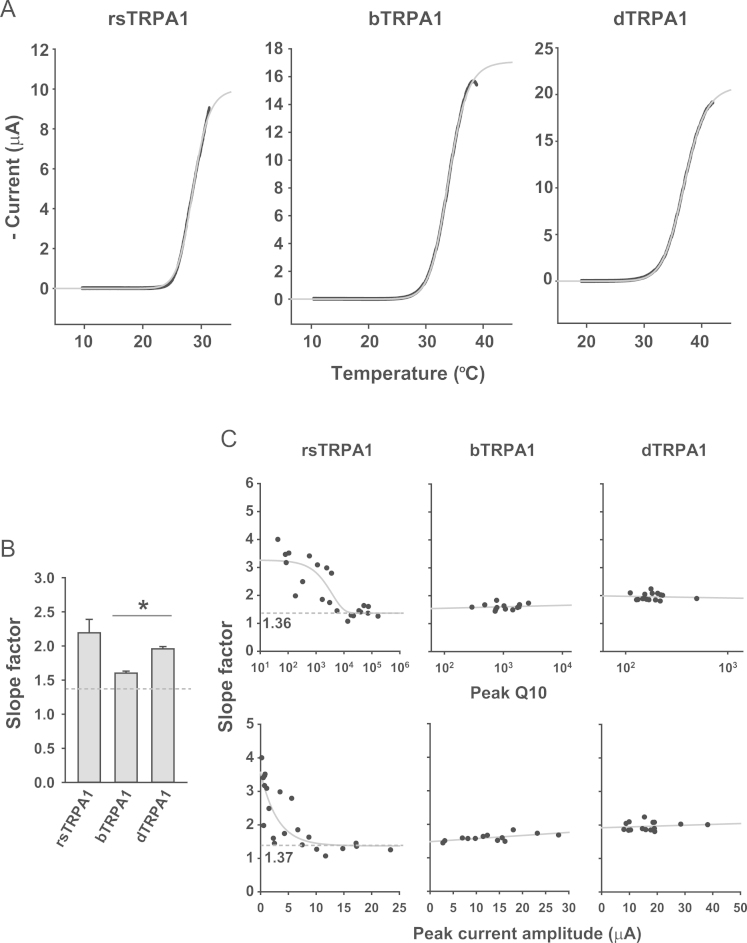
Thermal sensitivity comparison by means of the Boltzmann slope factor. (A) Temperature-evoked current traces of indicated TRPA1 were fitted to the Boltzmann equation with Sigmaplot12.0. Black traces represent the acquired current data at -60 mV. (B) The averages of slope factors from the three orthologues were presented. (C) Slope factors were plotted with either peak Q10 (Upper) or peak current amplitude (Lower) for indicated TRPA1s. The slope factors of rsTRPA1 could be fitted to the equation of exponential decay, *y*=ae^−bx^+c. *a*=1.9, *b*=0.00027 and *c*=1.36 for peak Q10 (Upper). *a*=2.2, *b*=0.33 and *c*=1.37 for amplitude (Lower). *: *p*<0.05. ANOVA Tukey test.

**Fig. 3:**
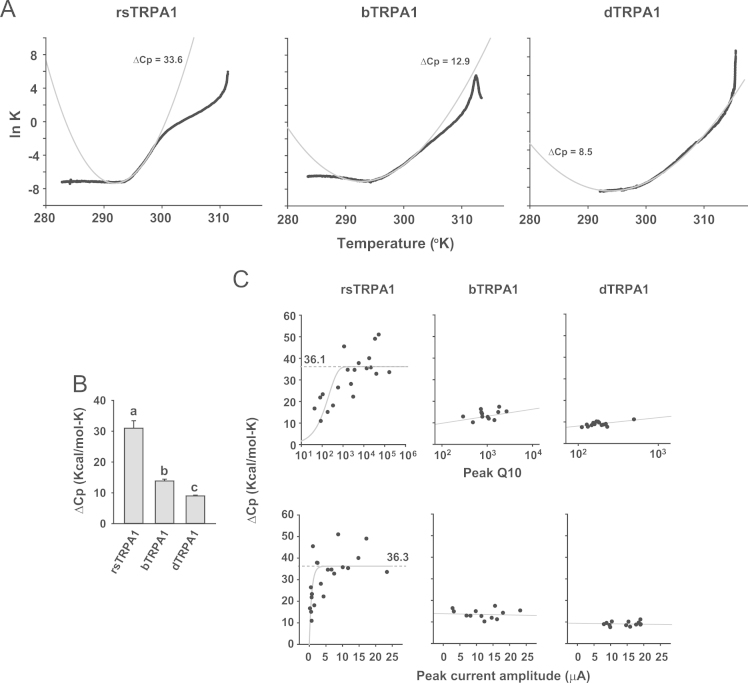
Thermal sensitivity comparison by means of the molar heat capacity change. (A) Temperature-evoked current traces of indicated TRPA1 were fitted to the ln K−Δ*Cp* equation with Sigmaplot 12.0 [7]. (B) The averages of Δ*Cp*׳s from the three orthologues were presented. (C) Δ*Cp*’s were plotted with either peak Q10 (Upper) or peak current amplitude (Lower) for indicated TRPA1s. Δ*Cp*’s of rsTRPA1 could be fitted to the equation of exponential rise to maximum, *y*=*a*(1−e^−bx^). *a*=36.1 and *b*=0.0048 for peak Q10 (Upper). *a*=36.3 and *b*=1.4 for amplitude (Lower). Letters in B indicates significantly distinct groups with *p*<0.05. ANOVA Dunn’s method.

To properly appreciate the high thermal sensitivity of TRPA1s from infrared-sensing snakes such as rattlesnakes and boas [1] in comparison with *Drosophila melanogaster* TRPA1 [2], [3], a new analysis for temperature coefficient Q10 (fold increase of current upon 10 °C temperature shift) was recently devised and called “Q10 scanning” [1]. By reducing the noise level of the temperature-evoked current through Gaussian filtering and shortening the interval between the two temperatures of Q10 calculation, the Q10 scanning method was tuned for higher Q10 sensitivity and more precise estimation of temperature parameters as presented here (Fig. 1). As alternative ways of analyzing temperature sensitivity of TRPA1s, the thermally induced current traces were fitted to equations to acquire the Boltzmann slope factor (Fig. 2) and the molar heat capacity change (Fig. 3).

## 2 Experimental design, materials and methods

The temperature-evoked current traces were previously acquired [1] by conducting two-electrode voltage clamping [4], [5] on *Xenopus laevis* oocytes expressing each TRPA1. The current traces were data-reduced by the factor of 10 through replacing 10 data points with their average value, filtered by the Gaussian low pass filter at 1 Hz, and further smoothened by averaging neighboring 50 data points in order to minimize the noise level. For each *t*1 temperature, Q10 was obtained by its definition (Q10=(I2/I1)^10/(*t*2−*t*1)^) with the use of two current and temperature data points apart from each other by 20,100, and 200 data points corresponding to 0.1, 0.5, and 1 s or 0.05, 0.25, and 0.5 °C, respectively. The calculated Q10s were plotted as function of temperature *t*1. The peak Q10 was computed by averaging 100 data points flanking the maximum Q10. The temperature where Q10 starts to increase or which produces the maximum Q10 was referred to as deflection temperature (*Td*) or peak temperature (*Tp*), respectively. *Td* and *Tp* were compared with the Arrhenius activation threshold temperature (*T*th) [6].

To obtain the Boltzmann slope factors, traces were fitted to the Boltzmann equation using Sigmaplot12.0. To acquire the change in molar heat capacity Δ*C_p_*, the temperature-evoked currents were transformed to traces of ln*K* by plotting ln I/(I_max_−I) as function of temperature, assuming for simple comparative analyses that I_max_ (peak current amplitude) is obtained with open probability of 1. The steepest part of the ln K graph was fitted with Sigmaplot 12.0 to the equation of “ln*K*=Δ*S*°_0_/*R*–Δ*C_p_*[1−*T*_0_/*T*+ln(T_0_/*T*)]/*R*” as function of *T* (temperature in Kelvin) to inquire the change of the molar heat capacity [7].
